# A Text-Book of Legal Medicine and Toxicology

**Published:** 1904-09

**Authors:** 


### Reviews of Books.

**A Text-book of Legal Medicine and Toxicology.** Edited by FREDERICK PETERSON, M.D., and WALTER S. HAINES, M.D. Vol. II. Pp. 825. London: W. B. Saunders & Co. 1904.

This is the second volume of a work the first volume of which was noticed by us some time ago. It is issued on the same principle as the former volume, and consists as before of a number of monographs by different writers. The volumes form a comprehensive survey of forensic medicine and toxicology. The greater part of this volume is occupied with toxicology, which has eight parts to itself. The first part deals with the malingering of impostors, prisoners, and feigned disease; the legal aspect of pregnancy, legitimacy, abortion, &c. Malpraxis is also referred to. The medico-legal aspect of the Röntgen Rays has a useful chapter; questions have already arisen in America as to the advisability of radiographs as evidence and their interpretation. The American laws relating to the insane and their position in the different States, is very fully dealt with.

The second part contains the poisons, and amongst other chapters one on food poisoning is of much use to-day, with our improved knowledge of ptomaines and proteid-toxins. A chapter up to date is given upon blood and blood stains, on seminal stains, hairs, and upon death from mechanical irritants, such as powdered glass. A concluding chapter is added on the responsibility of pharmacists in compounding prescriptions, selling poisons, and substituting other drugs than those prescribed. In America it appears the qualifications of pharmacists are left wholly to the Boards and Commissioners of Pharmacy, and the sale of poisons is differently regulated in each State.

